# Respiratory-Gated MRgHIFU in Upper Abdomen Using an MR-Compatible In-Bore Digital Camera

**DOI:** 10.1155/2014/421726

**Published:** 2014-01-29

**Authors:** Vincent Auboiroux, Lorena Petrusca, Magalie Viallon, Arnaud Muller, Sylvain Terraz, Romain Breguet, Xavier Montet, Christoph D. Becker, Rares Salomir

**Affiliations:** ^1^Faculty of Medicine, University of Geneva, 1211 Geneva, Switzerland; ^2^Clinatec/LETI/CEA, 38054 Grenoble, France; ^3^Department of Radiology, University Hospitals of Geneva, 1211 Geneva, Switzerland; ^4^Radiology Department, Lyon-Sud Hospital, University Hospitals of Lyon, 69495 Pierre Bénite, France; ^5^CREATIS, CNRS UMR5220, INSERM U1044, University of Lyon, INSA Lyon, 69621 Villeurbanne, France

## Abstract

*Objective*. To demonstrate the technical feasibility and the potential interest of using a digital optical camera inside the MR magnet bore for monitoring the breathing cycle and subsequently gating the PRFS MR thermometry, MR-ARFI measurement, and MRgHIFU sonication in the upper abdomen. *Materials and Methods*. A digital camera was reengineered to remove its magnetic parts and was further equipped with a 7 m long USB cable. The system was electromagnetically shielded and operated inside the bore of a closed 3T clinical scanner. Suitable triggers were generated based on real-time motion analysis of the images produced by the camera (resolution 640 × 480 pixels, 30 fps). Respiratory-gated MR-ARFI prepared MRgHIFU ablation was performed in the kidney and liver of two sheep *in vivo*, under general anaesthesia and ventilator-driven forced breathing. *Results*. The optical device demonstrated very good MR compatibility. The current setup permitted the acquisition of motion artefact-free and high resolution MR 2D ARFI and multiplanar interleaved PRFS thermometry (average SNR 30 in liver and 56 in kidney). Microscopic histology indicated precise focal lesions with sharply delineated margins following the respiratory-gated HIFU sonications. *Conclusion*. The proof-of-concept for respiratory motion management in MRgHIFU using an in-bore digital camera has been validated *in vivo*.

## 1. Introduction

Extracorporeal high intensity focused ultrasound (HIFU) is the only known technology permitting the delivery of sharp patterns of thermal energy within profound tissues [1]. MRI guidance of HIFU demonstrated essential advantages in terms of preoperative visualisation of the target volume, *in situ* localisation of the focal point by MR-ARFI, near real-time multiplanar thermometry, and immediate posttreatment assessment of acute effects of the therapy [2, 3].

The motion of upper abdomen organs is an important challenge for extracorporeal therapy using high intensity focused ultrasound [4]. If not compensated for, organ motion may hamper the MRgHIFU procedures, resulting in loss of treatment efficiency and inaccurate temperature monitoring. The main physiological cause of generalised abdominal motion is breathing. However, localised and smaller amplitude motions may occur because of bowel peristaltism and heartbeat.

Several methods for motion management dedicated to MRgHIFU in the upper abdomen have been investigated. Self-breath-holding is the simplest method and is performed without general anaesthesia, since it consists of asking the patient to voluntarily achieve apnoea as reproducibly as possible [5]. The success of the method is highly patient-dependent and intraoperatory pain may limit its applicability. The passive breath-hold method is performed under general anaesthesia with mechanical ventilation [6] and thus does not require the patient's control. Breath-hold is more easily and reproducibly obtained by turning the respirator off. Even if a single elementary sonication is feasible during one apnoea, treatment times with this technique are reported to be as long as 4.5–7 hours.

HIFU sonication using repeated gating under continuous breathing consists of applying the acoustic energy in portions similar to strobe lights and firing each time that the target is in the same position. The principle involves monitoring the respiratory cycle in real-time and performing the sonications at a specific phase of the respiratory cycle, which is always identical, called the “gating window”; the position of the tumour is the same if no other movement occurs (i.e., abdominal wall movement, cough, etc.) A recent report using this technique in sheep liver is provided by [7].

Single lung ventilation consists of using a dual-lumen intubation tube allowing single ventilation of the lung on the opposite side to the treated organ, thus minimising motion of the contralateral abdominal organ. It has been clinically tested for both the liver and kidneys with ultrasound-guided HIFU [8] but cannot completely “freeze” or suppress the abdominal motion.

Active motion tracking with real-time focal point lock-and-follow of the target [9–11] is based on the principle of sonicating continuously (i.e., theoretically maintaining a 100% duty cycle), despite movement. Besides the rapid detection of motion, this method requires the focal point to be relocated dynamically according to target displacement with low latency. The sonication should be stopped when the target is out of the steering range of the transducer.

In this paper, we describe an improved solution to respiratory-gated MRgHIFU in the upper abdomen, using an MR-compatible in-bore digital camera to monitor the abdomen or thorax expansion during breathing.

The idea of using a camera located inside the tunnel of a closed-bore MR system has been suggested in the past for brain MRI applications. The subject's head motion was tracked using an analogical in-bore camera and a checkerboard marker attached to the forehead of the patient; such an approach is described by Forman et al. [12]. This report describes the use of a self-encoded marker with each feature on the pattern being augmented with a 2D barcode, tracked by a single analogue in-bore camera attached to the head MR-coil. Outside of the scanner room, the analogue video signal is converted into a digital signal using a frame grabber. Motion correction using this approach encompassed a rotation of 18° around the principle axis of the cylindrical phantom between two scans. After rigid registration of the resulting volumes, a maximum error of 0.39 mm and 0.15° in translation and rotation was measured, respectively. This technology has been previously used for the correction of fMRI data, but has not been used in the context of MRgHIFU, to the best of our knowledge. Moreover, digital signal transmission over long lines, especially in the presence of potential noise or RF interference, is intrinsically more stable than with analogue signals.

The advantages of optical-based gating of both Proton Resonance Frequency Shift (PRFS) MR thermometry and HIFU sonication in the abdomen are presented here, together with demonstrative examples of the achievable accuracy of motion “suppression” in terms of HIFU targeting in the kidneys and liver, accurate PRFS MR thermometry for monitoring thermal ablation, and MR-ARFI for *in situ* characterisation of the HIFU beam steering.

## 2. Material and Methods

### 2.1. MR-Compatible Digital Camera and Optical Image Processing

In this study, the MR-compatible digital camera was a commercially available USB2 device (Quickcam pro 9000, Logitech, set to resolution 640 × 480 pixels, 30 fps) that had been made MR-compatible via the removal of any magnetic parts and the addition of RF shielding. The maximum signalling rate for this USB standard is 480 Mbit/s, with effective throughput up to 35 MB/s or 280 Mbit/s. A multitiered electromagnetically (EM)-shielded cable proceeded through the Faraday cage via a waveguide and connected the digital device (operating inside the magnet bore) to the external computer (operating outside the magnet room). The shielding coating of the cable was connected to the Faraday cage and the DC voltage ground was separated from the shielding. Twisted pair conductors D+ and D− for transferring USB data and, respectively, voltage supply for the USB level (5 V) and DC ground were available inside the shielded cable. A USB hub using an external power source was inserted on the communication line. That USB hub was located outside the Faraday's cage and permitted to increase the output DC current capability of the voltage supply up to 2 A. Optionally, an additional and independent circuit of DC voltage (12 V, twisted pair voltage supply and DC ground) was available inside the shielded cable to feed a high power LED-based light source. This MR-compatible lighting system was operated simultaneously with the acquisition of images by the digital camera in order to appropriately illuminate the field of view.

**Figure 1:**
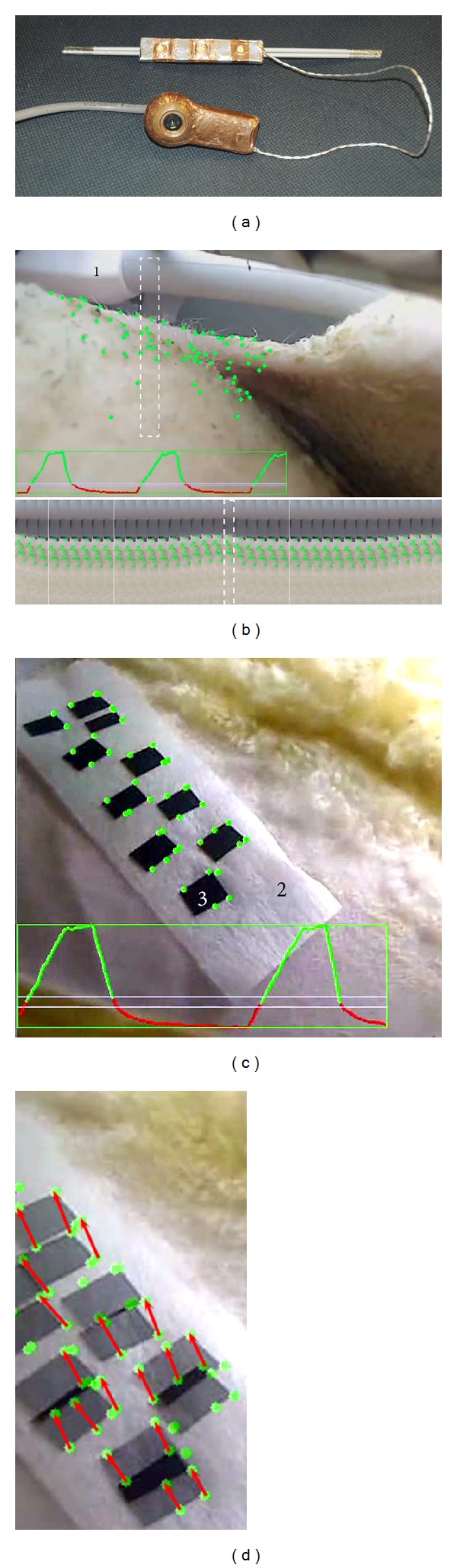
(a) Active MR-compatible USB-driven digital optical camera and the MR-compatible high power 3 LED lighting device. (b) Optical motion tracking experiment inside the MR bore on sheep abdomen, during forced breathing under general anaesthesia. Note the MR coil connector (1). The green dots are the automatically determined registration points. An exemplary breathing signal curve and a time-scrolled narrow vertical band from the image are shown. (c) A similar experiment, where a patch (2) was applied on the animal skin and high contrast markers were drawn (3). (d) Two overlapped frames from exhalation and inhalation phases, respectively, using a 50% transparency layer, same optical data as (c). The motion vectors of the representative optical registration points are drawn with red arrows.

The digital camera was EM-shielded by completely covering it with copper tape, except for a circular opening (approximately 2 mm in diameter) that is needed for the optical aperture. The copper tape was electrically connected to the shielding coating of the connection cable. A similar surface shielding was used for the illuminating LED system (see Figure 1(a)). For safety reasons, the digital camera was switched off during the preparation stage of the MR acquisition (frequency adjustment and shimming), in order to avoid exposure to high RF power peaks.

An optical camera can be mounted to a nonmagnetic orbital ring located in the magnet bore, or can be mechanically attached to the HIFU platform. The first solution was used here considering the field depth of our camera.

Suitable triggers based on motion analysis of the images generated by the camera were implemented using home written C++ code and OpenCV libraries (Open Source Computer Vision Library, http://www.opencv.org/) on a standard personal computer (Intel Core Duo P8700 CPU 2.53 GHz 2 GB RAM). Registration points were automatically set on sharp edge-features inside the user-defined optical region of interest (oROI) using an algorithm detecting the most prominent features, see Figures 1(b) and 1(c). This functionality was achieved by the “GoodFeaturesToTrack” routine from OpenCV, implementing the algorithm described by [13]. Once initialized, their displacement could be tracked using a calculation of the optical flow based on the iterative Lucas-Kanade method [14], the pyramids variant as described by [15]. The output data of the algorithm (see below) was initiated if at least 10 registration points were validated inside the user-defined oROI. If not, the oROI should be redefined or the image contrast improved. In the course of the procedure, some registration points could be automatically discarded if undergoing insufficient motion (less than 2 pixels), or if physically disappearing from the image. Tracking a larger number of points permitted noise-robust output signal; however, a single registration point was technically sufficient to generate a breathing curve. The optical data from the camera were processed in real-time (no processing queue) at 30 fps.

The output of the motion detecting algorithm was a respiration curve (see Figures 1(b) and 1(c)), which was used to determine the binary output status LOW/HIGH of a conventional DAC interface. The respiration curve was generated from the motion vector field of the registration points, which is illustrated in Figure 1(d). Here, the horizontal or vertical projection of the average displacement vector was calculated (equivalent, the 1D projection of the barycentre's displacement). Other metrics may be defined, for example, the average distance between the registration points and their actual barycentre. The output status was set to HIGH (TTL level) when the respiration curve fell below a threshold defined at 15% of the height of the dynamic peak-to-peak amplitude and was set to LOW (TTL level) when the respiration curve was above 25% of the height of the same amplitude. This binary signal was sent simultaneously (1) to the HIFU beam former, in order to dynamically switch the HIFU beam emission ON/OFF, and (2) to the sequence control processor of the magnetic resonance apparatus, in order to trigger the acquisition of magnetic resonance data; for example, MR thermometry or MR acoustic radiation force imaging (ARFI).

### 2.2. Animal Studies

The animal protocol was approved by the Geneva University Institutional Animal Care and Use Committee and by the Cantonal Veterinary Authority of Geneva. Two sheep (female, 25–35 kg bw) were included. Each animal was premedicated with ketamine (30 mg/kg; Pfizer, Zürich, Switzerland) and midazolam (0.2 mg/kg; Roche Pharma, Reinach, Switzerland). After placing an intravenous access using a jugular vein, the sheep were intubated and mechanically ventilated (Anaesthesia Delivery Unit [ADU] Plus Carestation; GE Healthcare, Madison, WI) using 6 m long ventilation tubing, and anaesthesia was maintained by continuous inhalation of 2% isoflurane (Abbott AG, Baar, Switzerland). The breathing rate was approximately 9–11 breaths/min. In the MR scanner, each animal was installed in a dedicated holder that allowed rigid translation and rotation of the animal body within the MR scanner, without touching the animal. Temperature regulation was provided using the continuous flow of warm water along the holder. Blood oxygen saturation, body temperature, and exhaled CO_2_ were monitored continuously. Immediately prior to HIFU sonication, an i.v. injection of 0.6 mg/kg atracurium (Labatec Pharma SA, Geneva, Switzerland) was administered to prevent any accidental muscular contraction during MRgHIFU ablation, thus ensuring a regular respiratory motion and accurate trigger/gating.

After ablation, the animals were awakened and followed-up for 7 days. Postoperative analgesia was administered (2 × 0.01 mg/kg per day; Buprenorphine; Essex Chemie AG, Lucerne, Switzerland) during 2 postoperative days. At day 7, the animals were followed up with MRI and then sacrificed. During MRI followup, contrast agent was injected intravenously (0.1 mmol/kg; Gadolinium, DOTAREM, Guerbet, France) and gadolinium-enhanced MR images were obtained to evaluate the size and position of the necrotic lesion, using the same T1-weighted 3D sequence (VIBE). The sonicated tissue was fixed in 4% formaldehyde and processed for macroscopic and microscopic analysis. Further details on the followup protocol and MR sequences can be found in [16].

### 2.3. MR-Guided HIFU Device and MR Data Acquisition

Focus ultrasound heating was produced using a 256-element phased array transducer (Imasonic, Besançon, France) with aperture *D* = 140 mm, natural focal length *R* = 130 mm, and a nominal frequency range from 968 kHz to 1049 kHz. The phased array was driven by a programmable 256-channel generator (Image Guided Therapy, Pessac-Bordeaux, France) with independent control of amplitude and phase per channel and was moved in the horizontal plane (*Ox* and *Oz* axes in the magnet frame) by a 2D positioning system with piezoelectric actuators (Image Guided Therapy, Pessac-Bordeaux, France). The −3 dB range of electronic steering of the HIFU device was ±15 mm lateral and ±25 mm axial.

MR-guided HIFU experiments were performed on a 3T whole body MRI scanner (Magnetom Trio a Tim system, Siemens AG, Germany), with a maximum gradient strength of 45 mT/m and slew rate of 200 T/m/s. A standard Siemens single loop receiver coil (11 cm diameter) with HIFU beam propagating through its aperture was used for MR signal acquisition in MRgHIFU renal ablation experiments, while for hepatic ablation two phased array coils wrapped around the animal were combined. A standard four-channel flex large coil (Siemens Medical Solutions, Erlangen, Germany) was placed on top of the abdomen, and a dedicated interventional three-channel coil (Clinical MR Solutions, Brookfield, WI, USA) with 14 × 16 cm^2^ aperture, enabling HIFU beam propagation, was placed under the animal. The scanner built-in adaptive coil combine algorithm was used.

The relative position of the animal in the HIFU transducer's frame of coordinates and the approximate position of the natural focal point within the kidney or the liver were visualised using a T1-weighted 3D fat-saturated gradient-echo sequence (VIBE) with the following parameters: TE (time of echo)/TR (time of repetition)/TA (time of acquisition)/FA (flip angle)/BW (band width) = 1.6 ms, 4 ms, 2.55 min, 10°, 650 Hz, and isotropic spatial resolution of 1.2 × 1.2 × 1.2 mm^3^. Further details on the planning stage are provided in [16].

A conventional radiofrequency-spoiled segmented EPI gradient echo sequence (GRE-EPI-MRT) was used for time-referenced PRFS MR thermometry [17, 18]. The main imaging parameters for this sequence were as follows: EPI factor 11; asymmetric echo train with phase direction partial Fourier 6/8; field of view (FOV) 128 mm × 128 mm; voxel size 1.0 × 1.0 × 5.0 mm^3^; 3 interleaved slices (coronal, sagittal, and transverse) acquired per breathing cycle; TR/TE/FA, 50 to 70 ms/ms/15 degrees; BW 738 hertz per pixel; echo spacing, 1.66 milliseconds; and TA per measurement 2.5 s. Spectrally selective 1-2-1 pulses were used to suppress lipid signals. Neither rectangular FOV nor parallel imaging was used.

The segmented GRE-EPI sequence was modified by adding bipolar motion encoding gradient (MEG) for single-slice acoustic radiation force imaging (MR-ARFI) [19]. The main imaging parameters of the MR-ARFI sequence were as follows: EPI factor, 11; asymmetric echo train with phase direction partial Fourier, 6/8; FOV 128 × 128 mm; voxel size, 1 × 1 × 5.0 mm; TR/TE/FA, 90 ms/16 ms/15 degrees; BW, 738 hertz per pixel; echo spacing, 1.66 milliseconds; image acquisition time, 1.05 seconds; total duration of the bipolar MEG, 8 ms; time shift between the HIFU pulse onset and the positive lobe MEG onset, 2.5 milliseconds.

The MR-compatible digital camera monitored the expansion of the abdomen during breathing. The image stream was processed in real-time and the output was used to trigger the MR-ARFI or MR thermometry and to gate the HIFU sonication during the quiet phase of exhalation, as previously described. A user-defined delay was available between the raising edge of the trigger signal and the effective starting point of the MR measurement. The focal point was positioned either in the sheep kidney (animal number 1) or in the sheep liver for intercostal sonication (animal number 2). The MR-ARFI was performed as described in [19], using pulsed HIFU (typically 5.5 ms pulse length, 240 acoustic watt) synchronised with bipolar motion encoding MR gradients (MEG). For ablative sonication, the applied peak power was 240 acoustic W and the sonication window covered 5 to 10 respiration cycles (approximately 30 s–60 s). The average duty cycle of the gated sonication was 33 to 65%, depending on the ventilator adjustments. HIFU sonication consisted of a single focus, or an iterated dithering of foci (one iteration per breathing gate, electronically steered pattern of foci, 8 mm in-plane size).

### 2.4. Noise Estimation in MR Images

In order to evaluate the noise increase in the MR images due to the simultaneous operation of the digital camera, a dynamic acquisition was performed using the GRE-EPI-MRT sequence. Dynamic measurements were acquired on a standard MR cylindrical phantom of approximately 2 liters. Thirty measurements were performed while the digital camera was active. Without discontinuing the MR acquisition, the camera was switched off and removed from the magnet bore and another 30 measurements were performed afterwards. The noise standard deviation was temporally determined pixelwise from the dynamic series of GRE-EPI-MRT magnitude data, within a large ROI (450 pixels) set central in the phantom. The noise measurements were performed for two different coil setups: (a) 11 cm diameter loop coil and (b) combination of 9 elements from the spine coil and body matrix coil used simultaneously.

## 3. Results

### 3.1. MR Compatibility

The MR-compatible digital camera was used at a distance of approximately 20 to 30 cm from the animal's body; in this situation, no MR image distortion nor off-resonance artefacts were detected within the FOV. Any minor shift in the GRE-EPI local phase was not found to be a problem as the position and orientation of the optical device were static through the acquisition time. The augmentation of the “white noise” in MR images due to the operation of the in-bore optical camera depended on the receive coil setup. As a general tendency, the 11 cm diameter loop coil was rather insensitive to any EM noise from the camera. In particular, when using the GRE-EPI-MRT sequence with that receiver loop coil, the noise increase was measured in the range 1 to 2%. Larger receiver coils, for instance, 9-element combination from spine and body matrix coils captured more EM noise from the optical device, yielding 5 to 7% higher noise SD in GRE-EPI-MRT data, as compared to the baseline situation without optical camera. Overall, active compatibility was demonstrated, as the level of additional noise in MR images was minimal to almost undetectable. On the other hand, the quality of the optical image delivered by the digital camera was found invariant, whether the MR acquisition was running or not. This effect was expected because of the digital processing of the optical information.

The maximum length of the transmission cable under the USB2 protocol compatible with the standard operation of the camera was 7 ± 0.2 m, measured from the powered USB hub to the digital camera. Time-out errors were generated by the driver software if the length of that transmission cable was increased beyond the limit. Overall, a 7 m distance is considered sufficient to enable out-of-room PC-based driving of the digital camera on the majority of MR system installations.

### 3.2. MR-ARFI Prepared MRgHIFU Volumetric Ablation in Sheep Kidney

**Figure 2:**
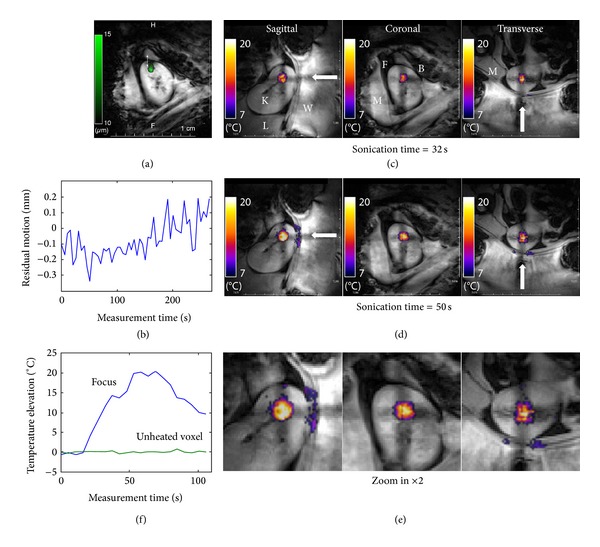
MR-ARFI prepared MRgHIFU thermoablation in sheep kidney. (a) Respiratory-gated MR-ARFI was performed according to [19] and permitted accurate visualisation of the focal point located in the inferior pole of the left kidney. A coronal slice is shown. FOV is 128 mm square. The scale of ARFI through-plane tissue displacement is provided at the left side in microns. (b) The position of the interface between the kidney and signal-suppressed perirenal fat was measured over 4 minutes of respiratory-gated MR-ARFI acquisition and demonstrated a very low level of residual motion in the submillimetre range. Because the tracked interface is smooth and orientated orthogonally to the main axis of motion (S/I), the actual resolution is higher than the intrinsic voxel size (1 mm in plane). (c) and (d) Multiplanar respiratory-gated MR thermometry during 8 mm pattern HIFU volumetric ablation in sheep kidney. The white arrows indicate the incidence direction of the HIFU beam. K: kidney, L: liver, W: coupling water, M: paravertebral muscles, B: bowel, and F: perirenal fat tissue. A temperature elevation colour scale is provided. FOV is 128 mm square. (e) Zoom-in of frame (d) around the focal zone. (f) Voxelwise temporal plot of temperature elevation, shown within the focal zone and for one unheated voxel located in the opposite pole of the treated kidney. Note the excellent baseline stability.

The current setup permitted the motion artefact-free acquisition of high resolution MR 2D ARFI and multiplanar interleaved PRFS thermometry for HIFU sonication in the kidney of sheep #1, as illustrated in Figure 2.

The position of anatomic landmarks in the vicinity of the kidney (e.g., renal capsule interface with perirenal fat), as seen in magnitude MR images, was found to be reproducible from one respiratory cycle to another, with less than half a millimetre drift over several minutes of acquisition (Figure 2(b)). After correcting for the slow drift, the standard deviation of the intercycle position of that landmark was found to be as low as 70 microns.

MR thermometry data in the kidney had a typical baseline SNR of 56 (range 40 to 80), corresponding to a temperature measurement average SD of 0.25°C. The main spatial variation of SNR in the kidney resulted from the histological structure of this organ with different T2* regions (cortex, medulla, pelvis). The 8 mm size foci pattern was sonicated iteratively (one iteration per breathing cycle), with a duty cycle of approximately 60% and applied acoustic power of 240 W yielding a temperature elevation in the sonicated focal area of more than 20°C after 50 s (Figures 2(d) and 2(e)). This result largely exceeds the lethal thermal dose threshold [20] and is considered very effective, given the high rate of blood flow in the kidney (1045 mL/min, [21]). The time-referenced PRFS thermometry baseline in unheated voxels (chosen in the opposite pole of the kidney relative to the focus position) was remarkably flat, with sole fluctuation owing to the “white” measurement noise (Figure 2(f)).

### 3.3. MR-ARFI Prepared MRgHIFU Intercostal Thermal Ablation in Sheep Liver

**Figure 3:**
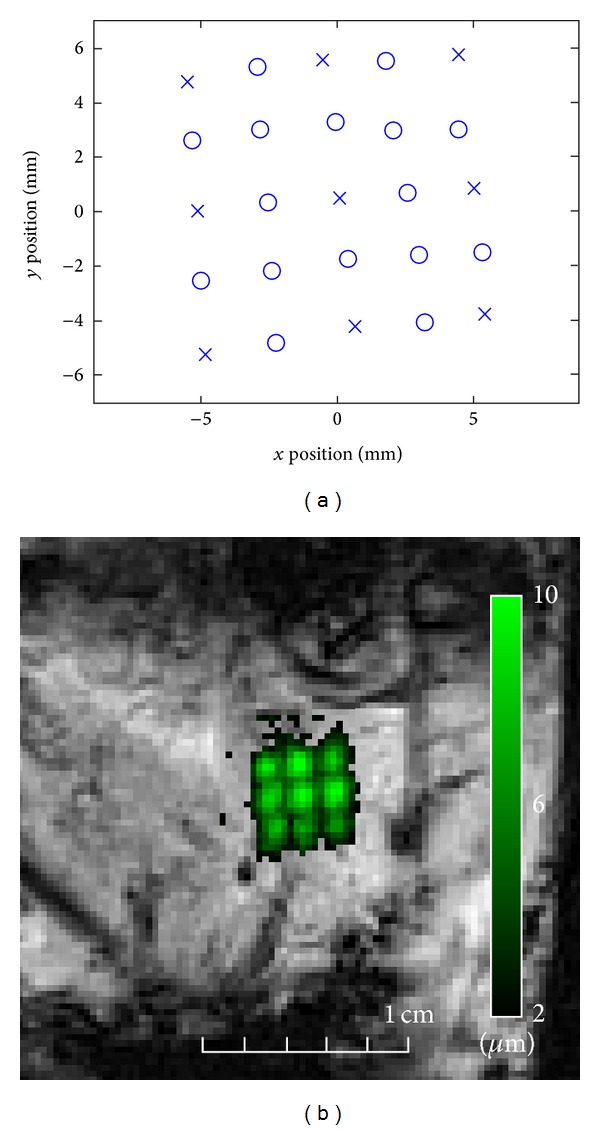
Respiratory-gated MR-ARFI mapping in sheep number 2 liver, evaluating the electronic HIFU beam steering accuracy *in situ*. (a) A 2D grid of 25 foci prescribed to be aligned with the MR reference frame and regularly spaced at 3 mm was sequentially sonicated using equal acoustic power HIFU pulses according to [19]. The measured positions of foci are drawn, indicating 5° counterclockwise rotation as compared to the MR reference frame. (b) The experimental positions of foci from a subgrid regularly spaced by 6 mm (corresponding to the ×–symbols in frame (a)) are shown overlaid on MR-ARFI magnitude data. A colour code is provided for the through-plane tissue displacement, together with a distance scale. A single sustained MR acquisition was performed over 15 minutes (approximately 75 respiration cycles), triggered by the breathing signal generated from the digital camera images.

The respiratory-gated MR-ARFI evaluation of the electronic steering in liver with intercostal sonication on a regular grid of foci (sheep #2) evidenced that the frame of coordinates used for the HIFU beam forming was slightly misaligned as compared to the MRI frame of coordinates. That misalignment consisted of a minor rotational offset of approximately 5 trigonometric degrees counterclockwise (Figure 3). This error is intrinsically minor but demonstrates the high precision of the MR-ARFI mapping that is achievable with the current setup. Furthermore, a measurable and smooth spatial modulation of the tissue displacement for different focus positions was observed at an equal applied power. This effect is jointly attributed to the steering apodisation of the phased array and to the tissue-specific variable response (e.g., variable shadowing of the ribs at different foci).

**Figure 4:**
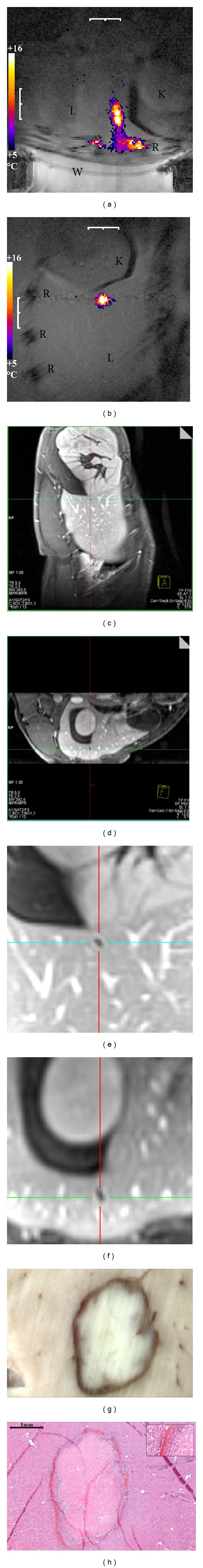
Thermal ablation with fixed focus, intercostal sonication in sheep liver and semichronic followup. K: kidney; L: liver; R: ribs; W: water; (a) and (b) transverse and, respectively, coronal MR thermometry at the sonication end point (40 sec time base), for the respiratory-gated sonication and MR acquisition. FOV is 128 mm square and a colour code for the measured temperature elevation is provided (range of +5°C to +16°C). (c) and (d) MPR sagittal and transverse, respectively, from isotropic 3D VIBE after contrast agent injection (early phase) at day 7 after treatment, shown FOV = 300 mm. (e) and (f) The frames (c) and (d), respectively, zoomed-in by a factor of 4. (g) Gross pathology after 3 weeks of formalin fixation of the liver tissue, shown FOV = 5.9 mm × 8.6 mm, sagittal slice. (h) HES microscopic staining, using the same distance scale as frame (f). Note the inset zoom-in by a factor of 3 (the upper right corner), demonstrating a very sharp transition margin between ablated and normal tissue, in the order of 100 microns.

The respiratory-gated MR thermometry data in liver parenchyma showed an average SNR of 30 (range of 25 to 40), corresponding to the average SD of thermometry of 0.46°C. The main spatial variation of SNR in liver was a result of the sensitivity field of the receiver coils. The respiratory-gated 40 s time base, fixed focus, high energy sonication yielded an accurately delineated elementary thermal lesion (Figure 4, sheep #2) of approximately 3 mm in-plane size and 5 mm along the incidence direction of the beam. The microscopic examination of the thermal lesion margins demonstrated a sharp transition between ablated tissue and normal aspect hepatic parenchyma at the scale of 100 microns.

## 4. Discussion

An advantage of the current approach is that the image data acquisition is contact-free and does not place any external obstacle in the HIFU beam entrance to the treatment site. In contrast to conventional mechanical sensors, such as an abdominal belt or a pressure cushion, which are operated in a user-dependent manner and may thus complicate abdominal interventional procedures, the reported approach is flexible and user independent and enables a large field of view for motion determination.

The available frame rate and resolution of commercial digital cameras are significantly higher than analogue standards. For example, cameras with up to 500 fps with a 1,280 × 1,024 pixel CMOS image sensor are commercially available as standard products. These features are advantageous for real-time, high resolution motion monitoring and correction. Moreover, digital devices are inherently more robust to EM noise and perturbation as compared to analogical transmission, even if the aforementioned RF shielding achieved might, in some instances, be suboptimal.

In contrast to belt or cushion-type respiratory sensors, which only provide a temporal curve, 2D or 3D dynamic images can be obtained here so that mapping of the surface motion is feasible, for example. Moreover, some patients may exhibit primarily thoracic breathing while others may exhibit predominantly abdominal breathing, thereby requiring the appropriate and accurate location of a mechanical sensor, which is not a critical factor for our approach.

Optical camera-based motion detection does not modify the patient's respiration patterns in any manner, in contrast to that which may occur with a mechanical sensor. Infrared (IR) cameras have a similar level of technological complexity and may, in principle, also be used in any situation when the IR spectral window would be advantageous.

The present method can provide the direct measurement of distances without the need for an indirect conversion from other parameters, such as pressure/volume or force/displacement, as is necessary with abdominal belts and pressure cushions. The calibration and response are essentially linear. The correction for any geometric distortion of the image can be easily achieved with a calibration board.

As compared to ultrasound-imaging-based monitoring of abdominal motion [9, 22], using the optical camera does not require an additional acoustic window alongside the already used HIFU acoustic window; also, most importantly, no interference is expected from the HIFU emission (electromagnetic or acoustic) into the optical images.

Since the practical implementation of the method was achieved using a commercial-grade camera, the method and apparatus may be economically implemented in clinical practice.

The operation of the digital camera inside the MR magnet bore yielded measurable additional noise in the MR images; however, using the standard GRE-EPI-MRT sequence jointly with 11 cm diameter loop coil the additional noise directly owning to the digital camera operation was less than 2% as compared to the baseline white noise level. Further improvement of the RF shielding engineering is possible whenever the additional noise would appear critical in some specific applications.

In perspective, this approach can be used to establish a correlation between multicamera stereoscopic images of the external patient surface and internal anatomical landmarks (similar to [23]), for active motion tracking during MRgHIFU similar to [10], using lookup table between optical images and tomographic MR data. Multiple cameras can be used to acquire the 3D shape of a body region, such as the abdomen, using stereoscopic reconstruction, as described in [24]. Correlation of respiratory motion between the external patient surface, determined from the optical data obtained with the digital camera and internal anatomical landmarks, obtained from fast dynamic MRI data [25], may be used for prospective motion compensation during MR-guided HIFU treatment. Alternatively, the external patient surface can be reconstructed using a single optical device by fringe projection profilometry, as described by [26].

## 5. Conclusion

A novel method for respiratory gating of MRgHIFU therapy was implemented and shown to be robust, accurate, and potentially suitable for future clinical application. The experimental proof-of-concept was validated with *in vivo* studies. Its advantages over the previously employed methods for achieving respiratory synchronisation of MRgHIFU in the upper abdomen have been highlighted.

## Supplementary Material

Illustration of optical data recorded by the in-bore digital camera during respiratory-gated MRgHIFU procedures in vivo. The motion vectors of sharp-edge features were used to generate a breathing signal curve. The native resolution and frame rate are shown.
